# 4-[(7-Fluoro­quinazolin-4-yl)­oxy]aniline

**DOI:** 10.1107/S1600536810053286

**Published:** 2010-12-24

**Authors:** Jing Jia, Guibin Wang, Dingqiang Lu

**Affiliations:** a School of Pharmaceutical Sciences, Nanjing University of Technology, No. 5 Xinmofan Road, Nanjing 210009, People’s Republic of China; b Jiangsu Provincial Institute of Materia Medica, Nanjing University of Technology, No. 26 Majia Street, Nanjing 210009, People’s Republic of China; c PRC DAYAOWAN Administration for Entry & Exit Inspection and Quarantine, Haiqingdao Foreign Area Development Zone, Dalian 116610, Liaoning Province, People’s Republic of China

## Abstract

In the mol­ecule of the title compound, C_14_H_10_FN_3_O, the bicyclic quinazoline system is effectively planar, with a mean deviation from planarity of 0.0140 (3) Å. The quinazoline heterocyclic system and the adjacent benzene ring make a dihedral angle of 85.73 (9)°. Two inter­molecular N—H⋯N hydrogen bonds contribute to the stability of the crystal structure. In addition, a weak π–π stacking inter­action [centroid–centroid distance = 3.902 (2) Å] is observed.

## Related literature

For general background to quinazolines, see: Labuda *et al.* (2009 ▶). Graves *et al.* (2002 ▶); For the preparation of the title compound, see: Zhang *et al.* (2010 ▶). For bond-length data, see: Allen *et al.* (1987 ▶).
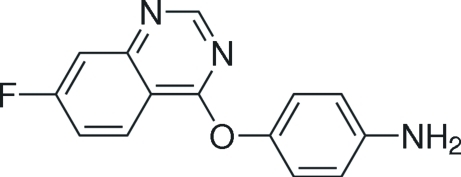


## Experimental

### 

#### Crystal data

C_14_H_10_FN_3_O
*M* *_r_* = 255.25
Orthorhombic, 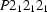

*a* = 8.0210 (16) Å
*b* = 8.3370 (17) Å
*c* = 17.562 (4) Å
*V* = 1174.4 (4) Å^3^
*Z* = 4
Mo *K*α radiation
μ = 0.11 mm^−1^
*T* = 293 K
0.30 × 0.20 × 0.10 mm

#### Data collection

Enraf–Nonius CAD-4 diffractometer
Absorption correction: ψ scan (North *et al.*, 1968 ▶) *T*_min_ = 0.969, *T*_max_ = 0.990
2351 measured reflections
1256 independent reflections
883 reflections with *I* > 2σ(*I*)
*R*_int_ = 0.082
3 standard reflections every 200 reflections  intensity decay: 1%

#### Refinement

*R*[*F* ^2^ > 2σ(*F* ^2^)] = 0.045
*wR*(*F* ^2^) = 0.110
*S* = 1.02
1256 reflections
172 parameters
H-atom parameters constrained
Δρ_max_ = 0.12 e Å^−3^
Δρ_min_ = −0.15 e Å^−3^

### 

Data collection: *CAD-4 EXPRESS* (Enraf–Nonius, 1994 ▶); cell refinement: *CAD-4 EXPRESS*; data reduction: *XCAD4* (Harms & Wocadlo, 1995 ▶); program(s) used to solve structure: *SHELXS97* (Sheldrick, 2008 ▶); program(s) used to refine structure: *SHELXL97* (Sheldrick, 2008 ▶); molecular graphics: *SHELXTL* (Sheldrick, 2008 ▶); software used to prepare material for publication: *SHELXL97* and *PLATON* (Spek, 2009 ▶).

## Supplementary Material

Crystal structure: contains datablocks global, I. DOI: 10.1107/S1600536810053286/zl2317sup1.cif

Structure factors: contains datablocks I. DOI: 10.1107/S1600536810053286/zl2317Isup2.hkl

Additional supplementary materials:  crystallographic information; 3D view; checkCIF report

## Figures and Tables

**Table 1:** Hydrogen-bond geometry (Å, °)

*D*—H⋯*A*	*D*—H	H⋯*A*	*D*⋯*A*	*D*—H⋯*A*
N1—H1*A*⋯N2^i^	0.89	2.67	3.408 (4)	142
N1—H1*B*⋯N3^ii^	0.89	2.38	3.205 (4)	154

Symmetry codes: (i) 
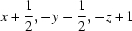
; (ii) 
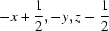
.

**Fig. 1.**
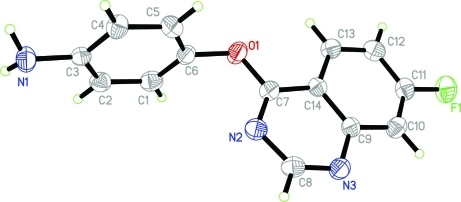
The molecular structure of the title molecule with the atom-numbering scheme. Displacement ellipsoids are drawn at the 30% probability level

**Fig. 2.**
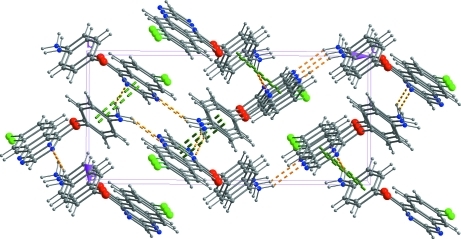
A partial packing diagram. Hydrogen bonds and weak π–π stacking interactions are shown as dashed lines.

## supplementary crystallographic information

### Comment

Quinazoline and its derivatives have been a research hotspot for a long time, owing to their significant role in the synthesis of some tyrosine protein kinase inhibitors and their potential anti-cancer activities (Labuda *et al.*, 2009; Graves *et al.*, 2002). As part of our studies into the synthesis of quinazoline derivatives, the title compound 4-[(4-benzenamine)yloxy]-7-fluoroquinazoline, which may be used as an intermediate towards some quinazoline derivatives, was synthesised. We report herein the crystal structure of the title compound.

In the molecule of the title compound, (Fig. 1), the bond lengths (Allen *et al.*, 1987) and angles are within normal ranges. The bicyclic quinazoline system is effectively planar with a mean deviation of only 0.0140 (3)Å. The dihedral angle between the benzene ring C1-C6 and the quinazoline ring system is 85.73 (9)°.

Two intermolecular N-H···N hydrogen bonds contribute to the stability of the molecular configuration and the packing of the molecules (Fig. 2 and Table 1). The crystal structure (Fig. 2) is also stabilized by a weak π–π stacking interaction with centroid–centroid separation of 3.902 (2) Å for Cg1···Cg2^i^ , where Cg1, Cg2 are the centroids of the rings N2/C7/C14/C9/N3/C8 and C1–C6, respectively [symmetry code: (i) -1/2+x, 1/2-y, 1-z].

### Experimental

The title compound was prepared by following a reported procedure (Zhang *et al.*, 2010). 4-Chloro-7-fluoroquinazoline (10 g, 54.77 mmol) was added to a mixture of dimethylformamide (100 ml), potassium tert-butoxide (6.15 g, 54.77 mmol) and 4-aminophenol (5.98 g, 54.77 mmol), and then heated to 343 K for 8 h. After cooling to room temperature, the reaction mixture was added to water (250 ml) and ethyl acetate (250 ml). The organic phase was collected and the water phase was extracted with ethyl acetate (250 ml). All the organic phases were combined and washed with brine (2× 250 ml). The organic phase was dried with anhydrous sodium sulfate for 6 h and then distilled (b.p. 313 K at 0.1 Mpa) and recrystallized from ethyl acetate, to give a total yield of 4-[(4-benzenamine)yloxy]-7-fluoroquinazoline of 81.0 % (11.32 g, 44.35 mmol). M.p. 353-355 K. ESI-MS(m/z): 256.1[M+H]^+^, 278.1[M+Na]^+^. ^1^H NMR (500 MHz, DMSO-d_6_) δ: 8.72 (s, 1 H, 8-H), 8.40 (dd, J_H-F_ = 6.0 Hz, J = 9.0 Hz, 1 H, 13-H), 7.7 (dd, J = 2.5 Hz, J_H-F_ = 10.0 Hz, 1 H, 10-H), 7.61 (td, J = 2.5, 9.0 Hz, J_H-F_ = 9.0 Hz, 1H, 12-H), 7.00 (d, J = 9.0 Hz, 1H, C1-H), 7.00 (d, J = 9.0 Hz, 1H, C5-H), 6.71 (d, J = 8.5 Hz, 1H, C2-H), 6.71 (d, J = 8.5 Hz, 1H, C4-H), 5.10(s, 2H, N1-H). ^13^C NMR(500 MHz, DMSO-d_6_) δ: 166.58 (C-7), 165.12 (d, J_C-F_ = 251.3 Hz, C-11), 155.17 (C-8), 152.64 (d, J_C-F_ = 13.8 Hz, C-9), 146.47 (C-6), 142.05 (C-3), 126.55 (d, J_C-F_ = 11.3 Hz, C-13), 121.89 (C-1), 121.89 (C-5), 117.38 (d, J_C-F_ = 25.0 Hz, C-12), 114.18 (C-2), 114.18 (C-4), 112.82 (C-14), 111.38 (d, J_C-F_ = 21.3 Hz, C-10). IR(KBr)(cm^-1^): ν_N-H_ (3420.80, 3327.69), ν_C__═__C-H_ (3077.53), ν_C__═__N_ (1628.11), δ_C__═__C-H_ (1609.25, 1575.87, 1509.07, 1459.02), ν_C-N_ (1286.23), ν_Ar-O_ (1248.06). UV-vis: λ_max_(CH_3_OH) nm (ε): 217.5 (40174), λ_max_ (0.1M HCl) nm (ε): 230.2 (30173), λ_max_(0.1M NaOH) nm (ε): 216.4 (16773).

Crystals of the title compound suitable for X-ray diffraction were grown from ethyl acetate.

### Refinement

The H atoms of the NH_2_ group were initially located from a Difference-Fourier map, but were then constrained to ride on their parent atom N1, with N-H = 0.89 Å, and U_iso_(H) = 1.2 U_eq_(N1) in the final stages of the refinement. The remaining H atoms were positioned geometrically with C-H = 0.93 and 0.98 Å for aromatic and methine H atoms, respectively, and constrained to ride on their parent atoms, with U_iso_(H) = 1.2 U_eq_(C). In the absence of any significant anomalous scattering, Friedel pairs were merged before the final refinement and the absolute structure was assigned arbitrarily.

### Crystal data

**Table:**

C_14_H_10_FN_3_O	*F*(000) = 528
*M**_r_* = 255.25	*D*_x_ = 1.444 Mg m^−^^3^
Orthorhombic, *P*2_1_2_1_2_1_	Mo *K*α radiation, λ = 0.71073 Å
Hall symbol: P 2ac 2ab	Cell parameters from 25 reflections
*a* = 8.0210 (16) Å	θ = 9.0–12.0°
*b* = 8.3370 (17) Å	µ = 0.11 mm^−^^1^
*c* = 17.562 (4) Å	*T* = 293 K
*V* = 1174.4 (4) Å^3^	Block, colorless
*Z* = 4	0.30 × 0.20 × 0.10 mm

### Data collection

**Table:**

Enraf–Nonius CAD-4 diffractometer	883 reflections with *I* > 2σ(*I*)
Radiation source: fine-focus sealed tube	*R*_int_ = 0.082
graphite	θ_max_ = 25.3°, θ_min_ = 2.3°
ω/2θ scans	*h* = −9→0
Absorption correction: ψ scan (North *et al.*, 1968)	*k* = −10→10
*T*_min_ = 0.969, *T*_max_ = 0.990	*l* = −21→0
2351 measured reflections	3 standard reflections every 200 reflections
1256 independent reflections	intensity decay: 1%

### Refinement

**Table:**

Refinement on *F*^2^	Primary atom site location: structure-invariant direct methods
Least-squares matrix: full	Secondary atom site location: difference Fourier map
*R*[*F*^2^ > 2σ(*F*^2^)] = 0.045	Hydrogen site location: inferred from neighbouring sites
*wR*(*F*^2^) = 0.110	H-atom parameters constrained
*S* = 1.02	*w* = 1/[σ^2^(*F*_o_^2^) + (0.046*P*)^2^] where *P* = (*F*_o_^2^ + 2*F*_c_^2^)/3
1256 reflections	(Δ/σ)_max_ < 0.001
172 parameters	Δρ_max_ = 0.12 e Å^−^^3^
0 restraints	Δρ_min_ = −0.15 e Å^−^^3^

### Special details

**Table:**

Geometry. All esds (except the esd in the dihedral angle between two l.s. planes) are estimated using the full covariance matrix. The cell esds are taken into account individually in the estimation of esds in distances, angles and torsion angles; correlations between esds in cell parameters are only used when they are defined by crystal symmetry. An approximate (isotropic) treatment of cell esds is used for estimating esds involving l.s. planes.
Refinement. Refinement of F^2^ against ALL reflections. The weighted R-factor wR and goodness of fit S are based on F^2^, conventional R-factors R are based on F, with F set to zero for negative F^2^. The threshold expression of F^2^ > 2sigma(F^2^) is used only for calculating R-factors(gt) etc. and is not relevant to the choice of reflections for refinement. R-factors based on F^2^ are statistically about twice as large as those based on F, and R- factors based on ALL data will be even larger.

### Fractional atomic coordinates and isotropic or equivalent isotropic displacement parameters (Å^2^)

**Table:**

	*x*	*y*	*z*	*U*_iso_*/*U*_eq_	
F1	0.3205 (3)	0.8351 (3)	0.76958 (14)	0.0778 (8)	
O1	0.4564 (4)	0.2636 (3)	0.54742 (14)	0.0600 (8)	
N1	0.5255 (5)	−0.3210 (3)	0.39680 (17)	0.0613 (9)	
H1A	0.6191	−0.3749	0.4060	0.074*	
H1B	0.5050	−0.3230	0.3470	0.074*	
N2	0.2795 (4)	0.1426 (3)	0.63318 (17)	0.0543 (9)	
N3	0.1694 (4)	0.2924 (4)	0.73803 (17)	0.0558 (9)	
C1	0.5944 (5)	0.0096 (4)	0.5327 (2)	0.0557 (10)	
H1C	0.6622	0.0345	0.5740	0.067*	
C2	0.6148 (5)	−0.1334 (4)	0.49402 (19)	0.0522 (9)	
H2B	0.6978	−0.2042	0.5093	0.063*	
C3	0.5145 (5)	−0.1730 (4)	0.43328 (19)	0.0449 (9)	
C4	0.3965 (5)	−0.0629 (4)	0.41001 (19)	0.0527 (10)	
H4A	0.3302	−0.0854	0.3679	0.063*	
C5	0.3757 (5)	0.0809 (4)	0.4487 (2)	0.0564 (10)	
H5A	0.2952	0.1540	0.4329	0.068*	
C6	0.4732 (5)	0.1138 (4)	0.50944 (18)	0.0472 (9)	
C7	0.3580 (5)	0.2687 (4)	0.60919 (19)	0.0467 (9)	
C8	0.1880 (5)	0.1638 (5)	0.6966 (2)	0.0593 (11)	
H8A	0.1297	0.0739	0.7132	0.071*	
C9	0.2538 (4)	0.4244 (4)	0.71251 (19)	0.0444 (8)	
C10	0.2435 (5)	0.5671 (4)	0.7550 (2)	0.0552 (10)	
H10A	0.1805	0.5725	0.7994	0.066*	
C11	0.3285 (6)	0.6964 (4)	0.7291 (2)	0.0540 (10)	
C12	0.4236 (5)	0.6976 (4)	0.6636 (2)	0.0580 (10)	
H12A	0.4787	0.7902	0.6481	0.070*	
C13	0.4348 (5)	0.5598 (4)	0.6223 (2)	0.0520 (10)	
H13A	0.4979	0.5577	0.5779	0.062*	
C14	0.3511 (4)	0.4204 (4)	0.64658 (18)	0.0420 (8)	

### Atomic displacement parameters (Å^2^)

**Table:**

	*U*^11^	*U*^22^	*U*^33^	*U*^12^	*U*^13^	*U*^23^
F1	0.0938 (19)	0.0589 (13)	0.0808 (16)	−0.0023 (15)	−0.0035 (15)	−0.0267 (12)
O1	0.0754 (19)	0.0483 (14)	0.0565 (14)	−0.0111 (16)	0.0223 (15)	−0.0100 (12)
N1	0.070 (2)	0.0483 (17)	0.0654 (19)	0.0034 (18)	0.0088 (19)	−0.0101 (15)
N2	0.0546 (19)	0.0488 (19)	0.059 (2)	−0.0069 (17)	0.0063 (18)	0.0018 (15)
N3	0.062 (2)	0.0521 (18)	0.0539 (18)	−0.0034 (18)	0.0099 (18)	−0.0034 (16)
C1	0.057 (2)	0.061 (2)	0.049 (2)	−0.007 (2)	−0.004 (2)	0.0017 (19)
C2	0.051 (2)	0.057 (2)	0.049 (2)	0.005 (2)	−0.0010 (19)	0.0061 (18)
C3	0.048 (2)	0.0434 (19)	0.0432 (19)	−0.0021 (19)	0.0097 (18)	0.0039 (16)
C4	0.050 (2)	0.059 (2)	0.049 (2)	−0.002 (2)	−0.008 (2)	−0.0106 (19)
C5	0.059 (2)	0.051 (2)	0.059 (2)	0.007 (2)	−0.003 (2)	0.005 (2)
C6	0.057 (2)	0.0423 (19)	0.0429 (19)	−0.0055 (19)	0.009 (2)	−0.0038 (17)
C7	0.047 (2)	0.049 (2)	0.0441 (19)	−0.002 (2)	0.0011 (19)	−0.0011 (17)
C8	0.059 (2)	0.055 (2)	0.064 (2)	−0.010 (2)	0.012 (2)	0.009 (2)
C9	0.0401 (19)	0.046 (2)	0.0473 (19)	0.0028 (18)	−0.0046 (18)	0.0004 (18)
C10	0.052 (2)	0.065 (2)	0.048 (2)	0.002 (2)	0.000 (2)	−0.0055 (19)
C11	0.057 (2)	0.050 (2)	0.054 (2)	0.001 (2)	−0.011 (2)	−0.0116 (19)
C12	0.058 (2)	0.050 (2)	0.066 (2)	−0.012 (2)	−0.004 (2)	0.002 (2)
C13	0.057 (2)	0.050 (2)	0.049 (2)	−0.007 (2)	−0.0003 (19)	−0.0032 (18)
C14	0.0399 (19)	0.0430 (18)	0.0431 (18)	0.0008 (17)	−0.0055 (17)	0.0003 (15)

### Geometric parameters (Å, °)

**Table:**

F1—C11	1.359 (4)	C4—C5	1.388 (4)
O1—C7	1.342 (4)	C4—H4A	0.9300
O1—C6	1.422 (4)	C5—C6	1.352 (5)
N1—C3	1.394 (4)	C5—H5A	0.9300
N1—H1A	0.8900	C7—C14	1.427 (4)
N1—H1B	0.8901	C8—H8A	0.9300
N2—C7	1.295 (4)	C9—C14	1.397 (5)
N2—C8	1.346 (4)	C9—C10	1.407 (5)
N3—C8	1.305 (5)	C10—C11	1.354 (5)
N3—C9	1.368 (4)	C10—H10A	0.9300
C1—C6	1.366 (5)	C11—C12	1.380 (5)
C1—C2	1.381 (5)	C12—C13	1.362 (5)
C1—H1C	0.9300	C12—H12A	0.9300
C2—C3	1.376 (5)	C13—C14	1.408 (4)
C2—H2B	0.9300	C13—H13A	0.9300
C3—C4	1.380 (5)		
			
C7—O1—C6	117.6 (3)	N2—C7—O1	121.5 (3)
C3—N1—H1A	114.6	N2—C7—C14	123.4 (3)
C3—N1—H1B	117.1	O1—C7—C14	115.0 (3)
H1A—N1—H1B	109.0	N3—C8—N2	129.2 (4)
C7—N2—C8	115.3 (3)	N3—C8—H8A	115.4
C8—N3—C9	115.0 (3)	N2—C8—H8A	115.4
C6—C1—C2	119.1 (4)	N3—C9—C14	121.9 (3)
C6—C1—H1C	120.5	N3—C9—C10	118.5 (3)
C2—C1—H1C	120.5	C14—C9—C10	119.6 (3)
C3—C2—C1	121.3 (4)	C11—C10—C9	117.7 (3)
C3—C2—H2B	119.4	C11—C10—H10A	121.2
C1—C2—H2B	119.4	C9—C10—H10A	121.2
C2—C3—C4	118.1 (3)	C10—C11—F1	118.5 (4)
C2—C3—N1	122.2 (4)	C10—C11—C12	124.4 (3)
C4—C3—N1	119.7 (3)	F1—C11—C12	117.1 (3)
C3—C4—C5	120.8 (3)	C13—C12—C11	118.3 (3)
C3—C4—H4A	119.6	C13—C12—H12A	120.9
C5—C4—H4A	119.6	C11—C12—H12A	120.9
C6—C5—C4	119.5 (4)	C12—C13—C14	120.2 (3)
C6—C5—H5A	120.3	C12—C13—H13A	119.9
C4—C5—H5A	120.3	C14—C13—H13A	119.9
C5—C6—C1	121.2 (3)	C9—C14—C13	119.8 (3)
C5—C6—O1	119.6 (3)	C9—C14—C7	115.1 (3)
C1—C6—O1	119.1 (3)	C13—C14—C7	125.0 (3)
			
C6—C1—C2—C3	−0.6 (6)	C8—N3—C9—C10	−178.5 (4)
C1—C2—C3—C4	2.4 (5)	N3—C9—C10—C11	179.7 (3)
C1—C2—C3—N1	−175.6 (4)	C14—C9—C10—C11	0.7 (5)
C2—C3—C4—C5	−2.4 (5)	C9—C10—C11—F1	−179.8 (4)
N1—C3—C4—C5	175.6 (4)	C9—C10—C11—C12	0.4 (6)
C3—C4—C5—C6	0.6 (6)	C10—C11—C12—C13	−0.6 (6)
C4—C5—C6—C1	1.3 (5)	F1—C11—C12—C13	179.5 (4)
C4—C5—C6—O1	177.7 (3)	C11—C12—C13—C14	−0.2 (6)
C2—C1—C6—C5	−1.3 (5)	N3—C9—C14—C13	179.5 (3)
C2—C1—C6—O1	−177.7 (3)	C10—C9—C14—C13	−1.5 (5)
C7—O1—C6—C5	95.7 (4)	N3—C9—C14—C7	−1.2 (5)
C7—O1—C6—C1	−87.9 (4)	C10—C9—C14—C7	177.8 (3)
C8—N2—C7—O1	178.7 (3)	C12—C13—C14—C9	1.2 (5)
C8—N2—C7—C14	0.5 (5)	C12—C13—C14—C7	−178.0 (4)
C6—O1—C7—N2	−0.7 (5)	N2—C7—C14—C9	0.7 (5)
C6—O1—C7—C14	177.7 (3)	O1—C7—C14—C9	−177.7 (3)
C9—N3—C8—N2	0.8 (6)	N2—C7—C14—C13	179.9 (3)
C7—N2—C8—N3	−1.3 (6)	O1—C7—C14—C13	1.5 (5)
C8—N3—C9—C14	0.5 (5)		

### Hydrogen-bond geometry (Å, °)

**Table:**

*D*—H···*A*	*D*—H	H···*A*	*D*···*A*	*D*—H···*A*
N1—H1A···N2^i^	0.89	2.67	3.408 (4)	142
N1—H1B···N3^ii^	0.89	2.38	3.205 (4)	154

Symmetry codes: (i) *x*+1/2, −*y*−1/2, −*z*+1; (ii) −*x*+1/2, −*y*, *z*−1/2.
